# Prevalence, Influencing Factors, and Clinical Characteristics of Cognitive Impairment in Elderly Patients With Schizophrenia

**DOI:** 10.3389/fpsyt.2022.910814

**Published:** 2022-06-06

**Authors:** Guojun Liu, Xiaoying Zhang, Xiaoning Huo, Wei Li

**Affiliations:** ^1^Department of Rehabilitation Medicine, Third People’s Hospital of Lanzhou, Lanzhou, China; ^2^Department of Geriatric Psychiatry, Shanghai Mental Health Center, Shanghai Jiao Tong University School of Medicine, Shanghai, China; ^3^Alzheimer’s Disease and Related Disorders Center, Shanghai Jiao Tong University, Shanghai, China

**Keywords:** elderly, chronic schizophrenia, cognitive impairment, hobby, MMSE

## Abstract

**Aims:**

The purpose of this study was to investigate the prevalence, influencing factors, and clinical characteristics of cognitive impairment in elderly patients with chronic schizophrenia.

**Materials and Methods:**

A total of 264 elderly patients with chronic schizophrenia and 156 normal controls were enrolled in the current study. The Mini-mental State Examination (MMSE) was used to assess their overall cognitive function, the Positive And Negative Syndrome Scale (PANSS) was used to assess their psychotic symptoms, the Geriatric Depression Scale (GDS) was used to assess their depressive symptoms, while Activity of Daily Living Scale (ADL) was used to assess their daily living ability.

**Results:**

The prevalence of cognitive impairment was 77.7% (205/264) in elderly patients with chronic schizophrenia, which was much higher than that [16.7% (26/156)] in normal controls. By using stepwise binary regression analysis, we found that hobbies (*p* < 0.001, OR = 0.224, 95% CI: 0.114–0.441) might be a protective factor for cognitive impairment, and this relationship remained statistically significant after adjusting for total scores of GDS, ADL and PANSS (model*^b^*) (*p* = 0.016, OR = 0.406, 95% CI: 0.195–0.847). Compared with individuals without cognitive impairment, individuals with cognitive impairment tend to have more depression and psychiatric symptoms as well as worse activities of daily living (*p* < 0.05). Through linear regression analysis of the mediating model, we found that hobbies may improve cognitive function by improving psychiatric symptoms, and play a partial mediating role (*B* = −4.789, *p* < 0.001).

**Conclusion:**

Cognitive impairment is a very prominent problem in elderly patients with chronic schizophrenia. Elderly schizophrenia patients with cognitive impairment tended to have more depressive mood, more psychotic symptoms and worse activities of daily living. Hobbies will help prevent cognitive impairment in elderly patients with schizophrenia and may improve their cognitive function by influencing psychiatric symptoms. Therefore, we should encourage elderly patients with chronic schizophrenia to develop their own hobbies. However, the above conclusion still need to be further verified, as we cannot exclude the effects of age and education.

## Introduction

Schizophrenia is characterized by widespread cognitive impairment, with varying degrees of impairment in all areas, such as attention, language, memory, and executive function, etc (1). Studies have shown that people diagnosed with schizophrenia will experience significant cognitive decline from the premorbid to the post onset period and worse cognitive function tends to predict worse outcomes (2). Moreover, cognitive performance is also one of the most important determinants of community functioning in patients with schizophrenia (3). Thus, cognition has been established as an important therapeutic target for improving functional outcomes in patients with schizophrenia (4).

Cumulative evidence suggests that people with schizophrenia are more likely to develop cognitive impairment than the general population. However, the data are scarce, and results vary widely (5, 6). For example, Peng et al. found that the prevalence of cognitive impairment among patients with early-stage schizophrenia was 84.7% (144/170), which was much higher than that in the general population (7). In Arunpongpaisal and Sangsirilak’s study, they found that the prevalence of cognitive impairment among patients with schizophrenia was 81.3% (8). In Keefe et al.’s study, they suggested that about 70% of patients with schizophrenia appeared to have moderate to severe cognitive impairments (9). While in Lennertz et al.’s study (10), only 33% of the patients with schizophrenia scored one standard deviation unit below the healthy control group in the general cognitive index. We speculate that the core reason for the differences in the above results may be the differences in survey methods and survey tools.

Neuropsychological studies show that the cognitive performance of patients with chronic schizophrenia will gradually decline with age and course of disease, and a considerable number of patients with chronic schizophrenia will have negative symptoms (11). Since age is the biggest influencing factor of cognitive function, different ages may also have a certain influence on relevant cognitive results (12, 13). In China, elderly patients with chronic schizophrenia are often hospitalized for a long time, and the closed environment often aggravates the cognitive impairment and hippocampal atrophy. Therefore, it is necessary to understand the prevalence and influencing factors of cognitive impairment in elderly patients with chronic schizophrenia, so as to provide targeted intervention and treatment.

Since there is no current study on cognitive impairment and its influencing factors in elderly patients with chronic schizophrenia, we will prepare to fill in the gaps in the above areas. In the current study, we recruited 264 elderly patients with schizophrenia who had been hospitalized for a long time. All the participants completed clinical evaluations, neuropsychological tests, and an investigation of cognitive-related factors. We hypothesized that long-term hospitalized elderly patients with chronic schizophrenia with cognitive impairment had more severe symptoms of cognitive impairment and more severe impairments in daily living than patients without cognitive impairment, and that hobbies might help prevent cognitive decline.

## Materials and Methods

### Participants

**FIGURE 1:**
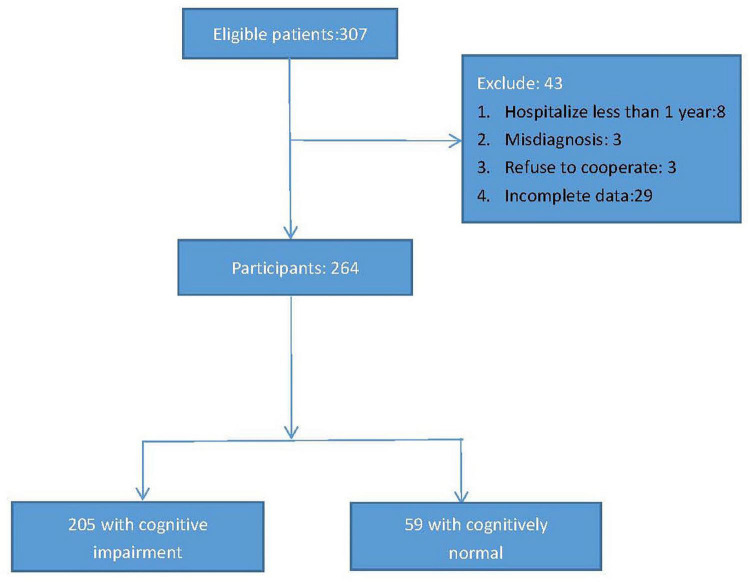
Research flow chart of whole study.

This cross-sectional study was conducted from 2021.1.1 to 2022.1.1, and elderly chronic patients with schizophrenia were recruited from Shanghai Mental Health Center and the third people’s hospital of Lanzhou. The inclusion criteria were as follows: (1) age 60 and above; (2) hospitalized for more than 1 year; (3) diagnosed with schizophrenia according to the International Classification of Diseases 10 diagnostic standards (ICD-10); (4) without obvious visual or hearing impairment (could not hear the assessor’s questions clearly or read the questionnaire clearly); (5) be willing to participate in the project. Exclusion criteria were as follows: (1) with serious or acute physical illness, such as myocardial infarction, cerebral hemorrhage, acute infection, cancer, etc; (2) suffering from other mental illnesses, such as bipolar disorder, depression, etc; (3) a diagnosis of alcohol/substance abuse; (4) be likely difficulty with participating in completing the survey. According to the above recruitment criteria, a total of 307 elderly chronic patients with schizophrenia were enrolled in the database, however, of the 307 participants, 8 had been hospitalized for less than a year, 3 had been diagnosed with bipolar disorder, 3 had refused to cooperate, 29 had incomplete data, so 264 patients with chronic schizophrenia were included in the final study (men/women = 124/140, average age: 67.15 ± 6.191, years). Figure 1 presents the research process.

All participants would undergo a complete clinical evaluation, neuropsychological evaluation, and physical examination. Through standardized questionnaires or medical records, we also collected their general demographic information (such as age, gender, years of education, Body mass index), daily life information (such as smoking, drinking alcohol, drinking tea, physical exercise, and hobbies), disease-related information (such as diabetes, hypertension), and medication status (types of antipsychotic drugs). Since antipsychotic drugs can affect the lipid metabolism of patients, in this study, we also collected their blood biochemical indicators, such as albumin, high density lipoprotein, low density lipoprotein, triglyceride and fasting blood glucose. Moreover, 156 age-matched and sex-matched elderly people from the community were also recruited as normal controls, and all the controls also met the previous inclusion and exclusion criteria, except for a diagnosis of schizophrenia.

The research protocol was approved by the Research Ethics Committees of the Shanghai Mental Health Center and the Third People’s Hospital of Lanzhou. Prior to the study, all participants received written informed consent. All research procedures were carried out in accordance with the principles of the Declaration of Helsinki.

### Cognitive Function Assessment

The mini-mental state examination (MMSE) is one of the most commonly used assessment tools in the field of geriatric cognition (14, 15). It mainly involves attention, recall, language, localization, registration, and calculation skills, and the score ranges from 0 to 30, with a lower score indicating poorer cognitive ability (16). It is very simple to use and the evaluation process only takes about 5 min, and has been proved effective in multiple populations (17, 18). Similarly, (MMSE) is also widely used in patients with schizophrenia, although there is a lack of normative data in this population (19). According to the conclusion of previous elderly community population studies, in our current study, the optimal cutoff scores of the MMSE for the groups aged ≤ 75 years old and education ≤ 6 years, aged > 75 years old and education ≤ 6 years, aged ≤ 75 years old and education > 6 years, aged > 75 years old and education > 6 years in screening for mild cognitive impairment were 26.5, 22.5, 28.5, and 26.5, respectively, and the optimal cutoff scores for dementia were 23.5, 19.5, 23.5, and 23.5, respectively (20). Subsequently, all individuals with cognitive impairment, including MCI and dementia, were assigned to the cognitively impaired group, while those with normal cognitive scores were assigned to the cognitively normal group.

### Daily Living Assessment

Cognitive function is closely related to the ability of daily living, and some individuals with cognitive impairment often show varying degrees of impairment in the ability of daily living. In the current study, activity of Daily Living Scale (ADL) was used to assess the daily functioning of elderly patients with chronic schizophrenia (21). The scale consists of two components; Basic self-care skills (BADL), such as self-feeding, dressing, and personal hygiene; Instrumental self-care (IADL), such as managing finances, leisure activities, and housework. Its score ranges from 14 to 64, with higher scores indicating greater loss of ability to perform daily tasks (22).

### Psychiatric Symptoms

The Positive and Negative Syndromes Scale (PANSS) is the most widely used symptom assessment scale in research and clinical trials for schizophrenia (23, 24). It consists of 30 items, and can provide a broad assessment of positive (7 items), negative (7 items), and general symptoms (16 items) (25). The PANSS is scored on the rating criteria of “past one week” and has been shown to be relatively sensitive to changes in symptoms (26). To date, PANSS is still considered the gold standard scale for symptom assessment in patients with schizophrenia (14).

### Depressive Symptoms

Since depressed mood can significantly affect cognitive performance, we also assessed participants’ depressive symptoms by using the Geriatric Depression Scale (GDS) (15). The GDS consists of 30 items with scores ranging from 0 to 30, with higher scores indicating more severe depressive symptoms (27, 28). It is able to assess a range of areas, ranging from emotion (e.g., crying, apathy, sadness) to cognition (e.g., helplessness, guilt, worthlessness) (29). Previous studies have demonstrated that GDS can effectively assess emotional symptoms in elderly patients with chronic schizophrenia (30).

### Blood Biochemical Test

All subjects stopped eating after 9 p.m and their peripheral blood was collected between 7 a.m. and 9 a.m. (the next morning). Blood indexes such as fasting blood glucose (FPG), low density lipoprotein (LDL), high density lipoprotein (HDL), cholesterol, and triglyceride were tested. All laboratory examination indexes were carried out in Shanghai Mental Health Laboratory of Blood biochemistry.

### Covariates

Through standardized questionnaires, we collected the subjects’ general demographic information, daily life information, and disease-related information. Information on smoking, alcohol consumption, exercise, and hobbies were collected based on self-report. Related questions took smoking as an example, as shown below. “Do you smoke?” If the answer was “yes,” we would further ask about the frequency and duration of smoking. Then, variables that differed between the cognitive impairment group and the non-cognitive impairment group were regarded as covariables.

## Statistical Analysis

Continuous variables were expressed as mean (standard deviation) and categorical variables as frequencies (%). Since none of the continuous variables conform to a normal distribution, Kolmogorov-Smirnov Z was performed between schizophrenia with cognitive impairment group and schizophrenia with cognitive normal group, while the Chi-square test was used to compare the categorical variables. Next, Different binary regression models were used to examine the association between cognitive impairment and the differential variables between the two groups. Model*^a^* controlled taking exercise and hobbies; Model*^b^* furtherly controlled other variables, such as the total score of ADL, GDS, and PANSS. Then a linear regression analysis (mediation model) was used to investigate the interaction between MMSE, hobbies, and neuropsychological tests. Two-tailed tests were performed at a significance level of *P* < 0.05, and all statistical analysis was performed using SPSS version 22.0 (IBM Corporation, Armonk, NY, United States).

## Results

### Prevalence of Cognitive Impairment in Elderly Patients With Schizophrenia

The prevalence of cognitive impairment in elderly patients with schizophrenia was 77.7% (205/264), while the prevalence of cognitive impairment in normal elderly was 16.7% (26/156). By using the chi square test, we found that the prevalence of cognitive impairment in elderly patients with schizophrenia was significantly higher (*X*^2^ = 147.349, *p* < 0.001) than that in normal controls.

### Comparison of General Demographic Data and Neuropsychological Tests Between the Two Groups (Elderly Patients With Schizophrenia With Cognitive Impairment vs. Elderly Patients With Schizophrenia Without Cognitive Impairment)

Patients with cognitive impairment had a lower proportion of take exercise and hobbies, lower MMSE scores, but higher GDS, ADL, and PANSS scores (*p* < 0.05) than patients without cognitive impairment. Table 1 presents the results.

**TABLE 1:** Demographic, clinical, and cognitive characteristics in chronic elderly schizophrenia with or without cognitive impairment.

Characteristics	Whole sample (*n* = 264)	Cognitive impairment (*n* = 205)	Cognitively normal (*n* = 59)	*p* value
Age, years (SD)	67.15 (6.62)	67.14 (6.95)	66.22 (5.25)	0.391
Education, years (SD)	8.15 (3.70)	7.91 (3.92)	8.97 (2.63)	0.308
Course of disease, years (SD)	36.34 (12.61)	36.61 (12.61)	35.40 (12.65)	0.288
The onset age, years (SD)	30.76 (12.60)	30.73 (12.74)	30.86 (12.24)	0.865
Albumin, g/L (SD)	40.51 (4.28)	40.46 (4.35)	40.69 (4.09)	0.991
Triglycerides, mmol/L (SD)	1.40 (0.81)	1.36 (0.76)	1.55 (0.96)	0.226
Total cholesterol, mmol/L (SD)	4.75 (1.00)	4.70 (0.98)	4.94 (1.06)	0.355
High density lipoprotein, mmol/L (SD)	1.33 (0.43)	1.34 (0.43)	1.31 (0.43)	0.595
Low density lipoprotein, mmol/L (SD)	2.76 (0.76)	2.73 (0.74)	2.87 (0.82)	0.603
Fasting plasma glucose, mmol/L (SD)	5.43 (1.28)	5.45 (1.31)	5.36 (1.18)	0.952
Body mass index, kg/m^2^ (SD)	23.94 (4.06)	23.86 (4.14)	24.25 (3.80)	0.523
Male, *n* (%)	140 (53.0)	108 (52.7)	32 (54.2)	0.883
Hypertension, *n* (%)	94 (35.6)	77 (37.6)	17 (28.8)	0.280
Diabetes, *n* (%)	65 (24.6)	51 (24.9)	14 (23.7)	1.000
Hyperlipidemia, *n* (%)	97 (36.7)	72 (35.1)	25 (42.4)	0.358
Smoker, *n* (%)	96 (36.4)	70 (34.1)	26 (44.1)	0.170
Drinker, *n* (%)	32 (12.1)	23 (12.1)	9 (15.3)	0.376
Take exercise, *n* (%)	95 (36.0)	66 (32.2)	29 (49.2)	0.021*
Hobby, *n* (%)	108 (40.9)	67 (32.7)	41 (69.5)	< 0.001
Atypical antipsychotics, *n* (%)	159 (62.4)	118 (59.9)	41 (70.7)	0.166
GDS	10.10 (5.89)	10.67 (5.96)	8.37 (5.37)	0.008*
MMSE	18.98 (7.88)	16.62 (7.40)	27.19 (1.09)	< 0.001*
ADL	27.19 (11.31)	29.63 (11.41)	19.03 (5.90)	< 0.001*
PANSS				
Positive symptom scale	12.13 (6.05)	12.31 (6.06)	11.53 (6.02)	0.237
Negative symptom scale	18.42 (8.37)	19.45 (8.57)	15.03 (6.71)	< 0.001*
General condition scale	32.92 (10.71)	33.86 (11.10)	29.81 (8.71)	0.003*
Total score scale	63.91 (21.39)	66.01 (21.71)	56.97 (18.85)	< 0.001*

*, p < 0.05; MMSE, Mini-mental State Examination; MoCA, Montreal Cognitive Assessment; GDS, Geriatric Depression Scale; ADL, Activity of Daily Living Scale; PANSS, Positive And Negative Syndrome Scale.

### Results of Stepwise Binary Regression Analysis and Receiver Operating Characteristic Curve

**FIGURE 2:**
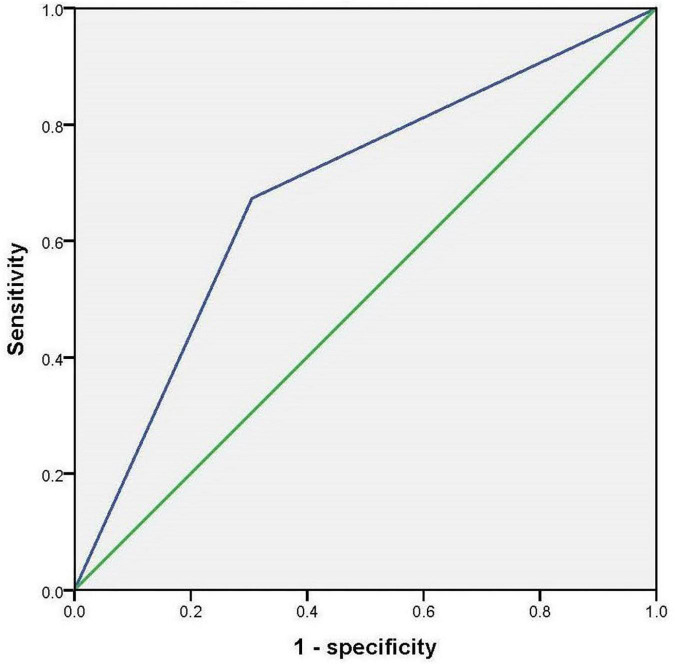
Receiver operating characteristic to curve to investigate the accuracy of hobbies in diagnosing cognitive impairment.

By using stepwise binary regression analysis, treating the presence or absence of cognitive impairment as the dependent variable, we found that hobbies (*p* < 0.001, OR = 0.224, 95% CI: 0.114–0.441) might be a protective factor for cognitive impairment (model*^a^*); This relationship remained statistically significant after adjusting for total scores of GDS, ADL and PANSS (model*^b^*) (*p* = 0.016, OR = 0.406, 95% CI: 0.195–0.847). Table 2 presents the results. The results of the receiver operating characteristic (ROC) curve showed that the area under the curve (AUC) for accuracy in diagnosing cognitive impairment using hobbies is 0.684 (*p* < 0.001, 95% confidence interval: 0.607–0.762). Figure 2 presents the results.

**TABLE 2:** Binary logistic regression analyses for factors related to cognitive impairment in chronic elderly schizophrenia.

Variables	B	S.E	Wals	df	*p*	OR	95% confidence interval
**Model^a^**							
Take exercise	–0.129	0.337	0.146	1	0.702	0.879	0.454–1.703
Hobby	–1.495	0.345	18.780	1	< 0.001*	0.224	0.114–0.441
**Model^b^**							
Take exercise	0.015	0.362	0.002	1	0.966	1.015	0.500–2.064
Hobby	–0.900	0.374	5.782	1	0.016*	0.406	0.195–0.847
GDS	–0.076	0.030	6.362	1	0.012*	0.927	0.873–0.983
ADL	–0.099	0.024	17.206	1	< 0.001*	0.906	0.864–0.949
PANSS	–0.011	0.011	1.034	1	0.309	0.989	0.969–1.010

*, p < 0.05; MMSE, Mini-mental State Examination; GDS, Geriatric depression scale; ADL, Activity of Daily Living Scale; PANSS, Positive And Negative Syndrome Scale.Model^a^ controlled take exercise and hobbies.Model^b^ continued to control GDS, ADL, and PANSS on the basis of Model^a^.

### Results of the Mediation Model of Linear Regression Analysis

**FIGURE 3:**
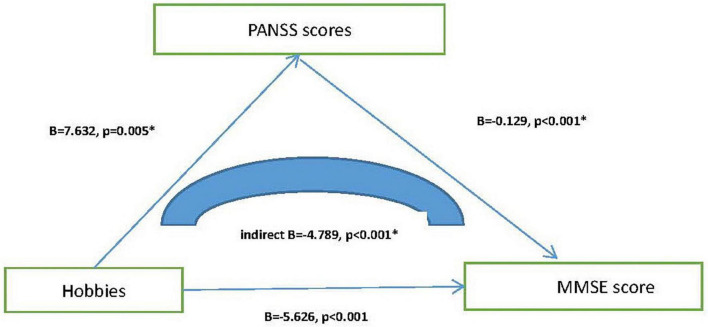
Mediating effect model among hobbies, PANSS, and MMSE score. *, *p* < 0.05; MMSE, Mini-mental State Examination; PANSS, Positive and Negative Syndrome Scale.

Next, we explore the relationship among MMSE total scores, hobbies and neuropsychological tests. The results of the mediation model of linear regression analysis show that hobbies affect MMSE scores by influencing PANSS total scores, and play a partial mediating role in this process (*p* < 0.001). However, there was no such association between MMSE, hobbies, and ADL/GDS. Figure 3 shows the results.

### Comparison of General Demographic Data and Neuropsychological Tests Between the Two Groups (Normal Elderly With Cognitive Impairment vs. Normal Elderly Without Cognitive Impairment)

To test whether these conclusion hold true in normal elderly people, we recruited 156 cognitively normal elderly people from the community, matched for age and sex with schizophrenia. However, the average years of education of normal elderly were significantly higher than that of patients with schizophrenia (9.77 ± 4.02 vs. 8.01 ± 3.72, *p* < 0.001). Compared with the elderly without cognitive impairment, the education level and MMSE score of the elderly with cognitive impairment were lower (*p* < 0.05), but there was no difference in age, gender, and other variables. The results of binary logistics regression analysis show that education is a protective factor of cognitive impairment in normal elderly (*p* = 0.001, OR = 0.848, 95% confidence interval: 0.766–0.939). Tables 3 4 show the results.

**TABLE 3:** Demographic, clinical, and cognitive characteristics in normal elderly with or without cognitive impairment.

Characteristics	Whole sample (*n* = 156)	Cognitive impairment (*n* = 26)	Cognitively normal (*n* = 130)	*p* value
Age, years (SD)	69.84 (7.61)	72.04 (9.19)	69.40 (7.21)	0.451
Education, years (SD)	9.77 (4.01)	7.38 (4.66)	10.25 (3.71)	0.022*
Triglycerides, mmol/L (SD)	1.93 (1.45)	1.55 (1.15)	2.01 (1.50)	0.226
Total cholesterol, mmol/L (SD)	4.94 (1.08)	4.87 (1.00)	4.95 (1.05)	0.715
High density lipoprotein, mmol/L (SD)	1.19 (0.27)	1.25 (0.35)	1.17 (0.25)	0.152
Low density lipoprotein, mmol/L (SD)	2.93 (0.84)	3.02 (0.81)	2.91 (0.85)	0.560
Fasting plasma glucose, mmol/L (SD)	5.44 (1.45)	5.24 (1.29)	5.49 (1.49)	0.163
Body mass index, kg/m^2^ (SD)	24.15 (3.38)	23.30 (2.92)	24.33 (3.45)	0.484
Male, *n* (%)	61 (39.1)	8 (30.8)	53 (40.8)	0.386
Hypertension, *n* (%)	79 (50.6)	16 (61.5)	63 (48.5)	0.284
Diabetes, *n* (%)	15 (9.6)	2 (7.7)	13 (10.0)	1.000
Hyperlipidemia, *n* (%)	26 (18.1)	6 (25.0)	20 (16.7)	0.297
Smoker, *n* (%)	35 (22.4)	5 (19.2)	30 (23.1)	0.800
Drinker, *n* (%)	30 (19.4)	3 (11.5)	27 (20.9)	0.414
Take exercise, *n* (%)	100 (65.8)	19 (73.1)	81 (64.3)	0.498
Hobby, *n* (%)	90 (60.4)	17 (65.4)	73 (59.3)	0.662
GDS	5.41 (4.34)	5.31 (4.48)	5.43 (4.33)	0.895
MMSE	27.99 (2.27)	24.47 (2.26)	28.74 (1.37)	< 0.001*
ADL	14.29 (1.97)	14.88 (2.47)	14.18 (1.85)	0.095

*, p < 0.05; MMSE, Mini-mental State Examination; MoCA, Montreal Cognitive Assessment; GDS, Geriatric Depression Scale; ADL, Activity of Daily Living Scale.

**TABLE 4:** Binary logistic regression analyses for factors related to cognitive impairment in normal elderly.

Variables	B	S.E	Wals	df	*p*	OR	95% confidence interval
Education	−0.165	0.052	10.106	1	0.001*	0.848	0.766–0.939

*, p < 0.05.

## Discussion

In the current study, we investigated the prevalence and influencing factors of cognitive impairment in elderly patients with chronic schizophrenia, and found that (1) the prevalence of cognitive impairment in elderly patients with chronic schizophrenia was 77.7%, which was much higher than that [16.7% (26/156)] in normal controls; (2) hobbies (*p* = 0.016, OR = 0.406, 95% CI: 0.195–0.847) was an important protective factor of cognitive impairment; (3) elderly schizophrenia patients with cognitive impairment tended to have more depressive mood, more psychotic symptoms, and worse ability of daily living; (4) hobbies might affect MMSE scores by influencing PANSS total scores, and play a partial mediating role in this process (*p* < 0.001).

Cognitive impairment is one of the core symptoms of schizophrenia and is more predictive of prognosis than other psychiatric symptoms (31). Understanding the prevalence and influencing factors of cognitive impairment in elderly patients with schizophrenia can not only better understand the development and evolution of schizophrenia, but also provide evidence for early prevention and intervention. Previous studies have shown that about 50–70% of people with schizophrenia will develop moderate to severe cognitive impairment (9, 32, 33). In our current study, we have also reached similar conclusion (77.7%), suggesting that cognitive impairment is a very prominent problem in elderly patients with schizophrenia.

Previous studies have shown that healthy lifestyle, such as a reasonable diet, wide social relationship, proper intensity of mental activity and maintaining curiosity about new things, will help prevent cognitive decline (34). Similarly, having a hobby or two that involves mental activity may also help prevent mild cognitive impairment or dementia (35, 36). However, other studies have shown that hobbies do not protect against dementia or MCI, and the correlation is due to the decline in hobbies that occurs in the early stages of dementia or MCI (37). In our study, we found that having hobbies is an important protective factor for cognitive impairment in elderly patients with schizophrenia, independent of ability to daily live and depressive and psychotic symptoms. But we have to point out that we found no association between hobbies and cognitive impairment in the cohort of normal older adults. There were two reasons for the differences in these studies. First, the age of our elderly patients with schizophrenia was significantly younger than that of normal controls, while education is an important determinant of cognitive function. Second, hobbies might be a result of cognitive impairment rather than a cause, since many patients with schizophrenia with low MMSE scores may have less interest to the hobby initially. Therefore, the association between cognitive impairment and hobbies needs to be further verified.

There are several mechanisms that might explain why hobbies might help prevent cognitive decline. First, having hobbies can slow brain atrophy and strengthen the synaptic function so as to delay the occurrence of cognitive impairment (38). Second, hobbies can help prevent cognitive decline by keeping the hands, feet and brain in a state of constant activity, which helps maintain sharp insight and analytical skills (35). Third, having hobbies often means greater social involvement, which can help prevent depression and functional disability in individuals (39). Fourth, some special hobbies, such as music and so on, can increase the thickness of the motor and somatosensory areas of the brain (40). Finally, slightly higher engagement in intellectual activities is also associated with higher levels of acquired hippocampal neurogenesis (41). Therefore, we encourage elderly patients with chronic schizophrenia to have more educational hobbies, such as reading, reading newspapers, playing chess, playing computer, painting, etc (42, 43).

Finally, we explored the association among cognitive symptoms, depressive symptoms, psychotic symptoms, and daily living activities in elderly patients with schizophrenia. By using non-parametric tests, we found that schizophrenia patients with cognitive impairment tend to have higher GDS, PANSS, and ADL scores, suggesting that they have more depressive mood and psychotic symptoms as well as more severe impairment of activities of daily living. In fact, the above conclusion are not very difficult to understand, schizophrenia itself is a serious mental illness involving positive symptoms, negative symptoms, and cognitive symptoms, and the symptoms often interact with each other (44). More intriguingly, we found that hobbies may improve cognitive function by influencing psychiatric symptoms, and may act as a partial mediator. Since there have been no previous studies like this, we could not tell if our findings were consistent with those of others.

We acknowledge that our study has some limitations. First, this was a cross-sectional study and it was impossible to establish a cause-and-effect relationship between cognitive impairment and daily living or mental symptoms; Second, the use of scale to diagnose cognitive impairment is prone to deviation; Third, we did not specify which hobbies were most likely to prevent cognitive decline; In addition, the relatively small sample size also reduces the reliability of the study.

## Conclusion

Cognitive impairment is very common in elderly patients with schizophrenia, which not only affects their prognosis, but also affects their quality of life. Elderly schizophrenia patients with cognitive impairment tended to have more depressive mood, more psychotic symptoms, and worse activities of daily living. Hobbies might help prevent cognitive impairment in elderly patients with schizophrenia and might improve their cognitive function by influencing psychiatric symptoms. However, the associations between cognitive impairment and hobbies need to be further verified, as we could not exclude the effects of age and education.

## Data Availability Statement

The original contributions presented in the study are included in the article/supplementary material, further inquiries can be directed to the corresponding author.

## Ethics Statement

The studies involving human participants were reviewed and approved by Shanghai Mental Health Center and the Third People’s Hospital of Lanzhou. The patients/participants provided their written informed consent to participate in this study.

## Author Contributions

GL designed and wrote the manuscript. XH made statistics and diagrams. WL and XZ collected the data and provided fund support. All authors have made significant contributions to the work of the report, whether in concept, study design, execution, data acquisition, analysis, and interpretation, or in all of these areas; participated in the drafting, revision, or critical review of manuscript; gave final approval of the forthcoming edition; agreed on the journal to which the manuscript was submitted; and agreed to be responsible for all aspects of the work.

## Conflict of Interest

The authors declare that the research was conducted in the absence of any commercial or financial relationships that could be construed as a potential conflict of interest.

## Publisher’s Note

All claims expressed in this article are solely those of the authors and do not necessarily represent those of their affiliated organizations, or those of the publisher, the editors and the reviewers. Any product that may be evaluated in this article, or claim that may be made by its manufacturer, is not guaranteed or endorsed by the publisher.
